# CT in a Neonate with Nasal Obstruction

**DOI:** 10.5334/jbr-btr.1365

**Published:** 2017-07-27

**Authors:** Stephanie Vanden Bossche, Geert De Vos, Marc Lemmerling

**Affiliations:** 1 University Hospital Ghent, BE; 2 AZ Sint-Lucas Ghent, BE

**Keywords:** CT, dacryocystocoele, pediatric

A three-day-old girl was referred to the radiology department by the otorhinolaryngologist for work-up of nasal obstruction as she presented with loud nasal respiration and respiratory distress during feeding. Nasal endoscopy revealed a bilateral intranasal mass. CT of the sinonasal region showed a bilateral soft-tissue mass at the medial canthus, with bilateral widening of the nasolacrimal canal, and revealed the presence of a bilateral endonasal mass below the inferior turbinate (Figures 1, 2, 3). This triad of imaging findings is diagnostic for a congenital dacryocystocoele. Endonasal wide marsupialisation using a microdebrider was performed, restoring patency of the nasolacrimal apparatus and resolving the symptoms.

**Figure 1:**
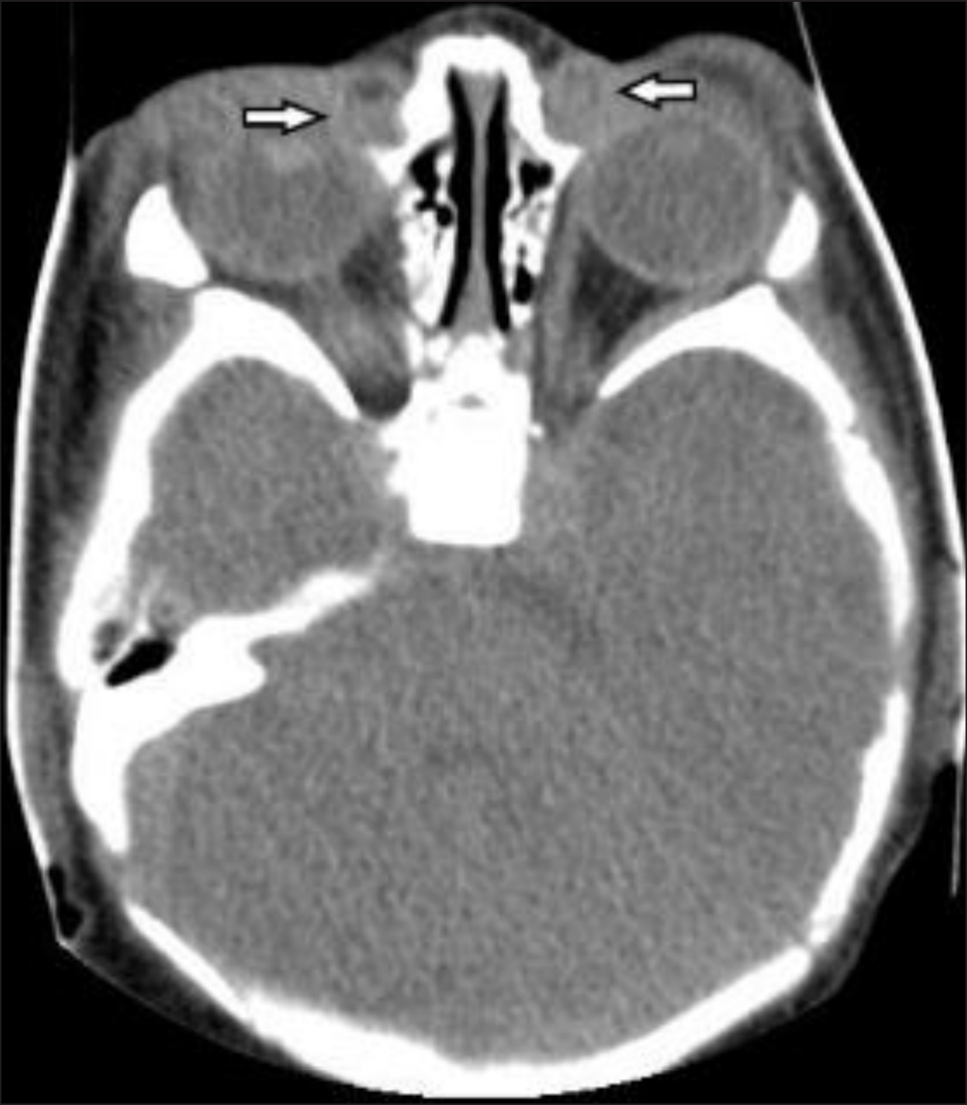
Axial CT image shows a bilateral soft tissue mass (arrows) at the level of the medial canthus, which is formed by the dilated lacrimal sac.

**Figure 2:**
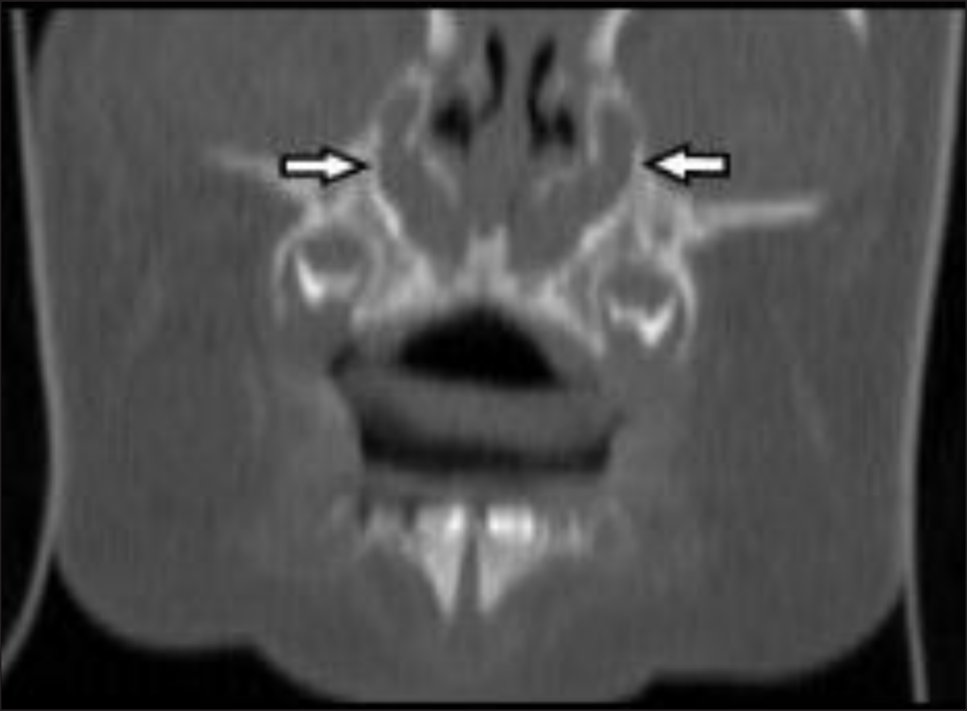
On the coronal reformed CT image in bone window, the dilation of the nasolacrimal duct can be appreciated.

**Figure 3:**
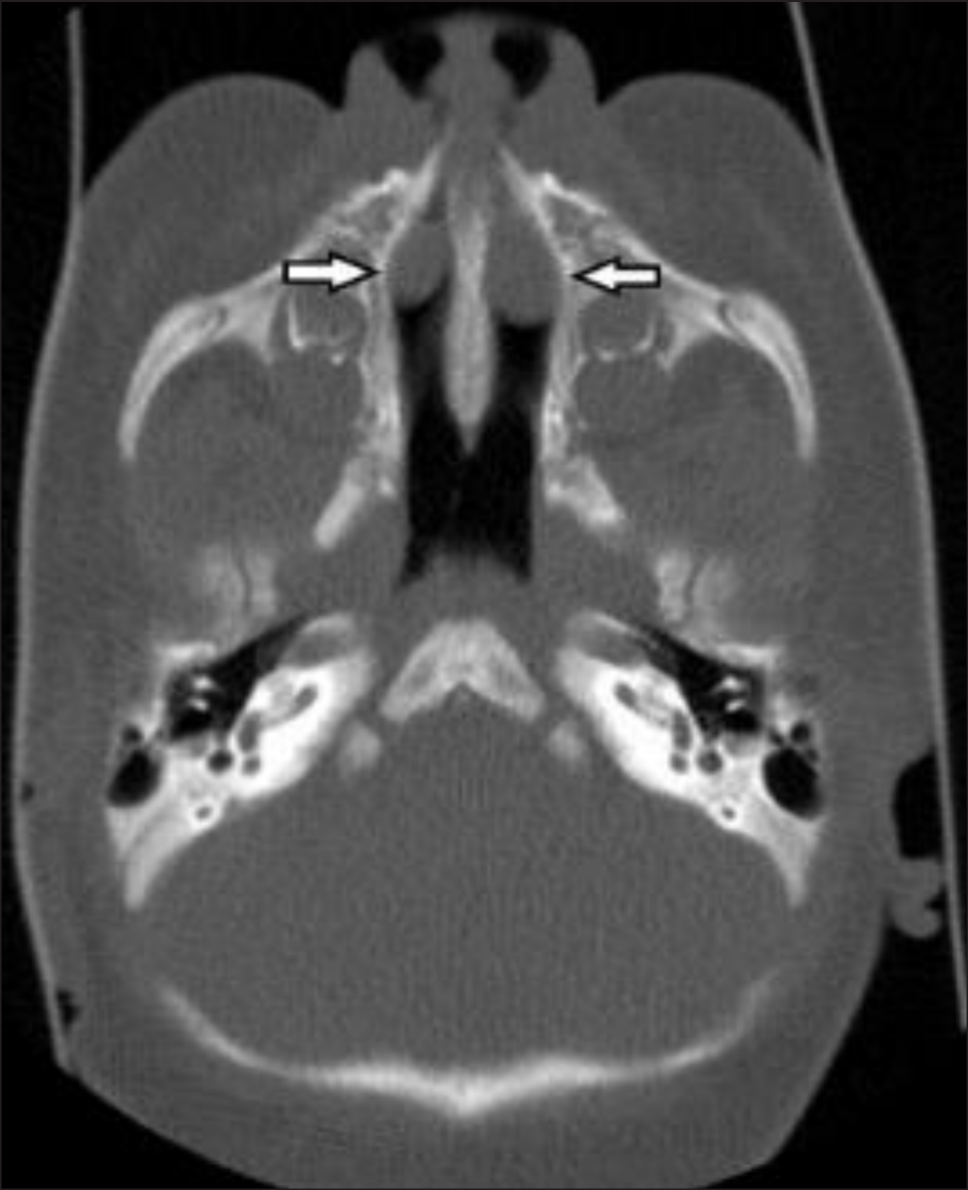
Axial CT image in bone window at the level of the nasal cavity shows the bilateral anterior intranasal mass, compatible with a bilateral dacryocystocele.

## Comment

The nasolacrimal apparatus is formed from an epithelial cord, which is canalized in craniocaudal direction from the third to the seventh month in utero. When canalization is incomplete at the caudal end a Hasner membrane persists, causing obstruction of the common congenital nasolacrimal duct, present in 30 percent of the infants. This membrane usually spontaneously ruptures during the first year of life, presumably due to an increased intraluminal pressure caused by crying, breathing and tear production, leading to the formation of the Hasner valve.

A dacryocystocoele is a rare variant of congenital nasolacrimal duct obstruction, occurring in 0.1 percent of cases, and is seen bilaterally in 25 percent of the patients. It is the second most common cause of nasal obstruction in neonates, after choanal atresia. Patients with a dacryocystocoele have a distal obstruction as described above, as well as a proximal functional obstruction caused by a ball-valve effect at the valve of Rosenmuller (between the common canaliculus and the lacrimal sac). Proximally, the continuous fluid filling of the nasolacrimal system causes distension of the nasolacrimal sac, blocking the canaliculus, which leads to the appearance of a firm blue swelling below the medial canthus. Distally, the mucus retention leads to ballooning of the Hasner membrane in the nasal cavity, appearing as an intranasal cyst.

CT is the imaging modality of choice to illustrate the classical triad of a cystic mass at the medial canthus, a dilated nasolacrimal duct and a cystic mass below the inferior nasal turbinate. Other anterior endonasal tumors that should be considered in the differential diagnosis are the rare intranasal glioma and an encephalocele. A glioma most often presents as a unilateral solid mass at the level of the middle turbinate, whereas an encephalocele is located medially and is associated with a bone defect.

Since newborns are obligate nasal breathers a dacrycystocoele can eventually be life-threatening when the nasal obstruction leads to respiratory distress [1]. The most important complication however is dacryocystitis, especially when it evolves to preseptal cellulitis, orbital cellulitis, sepsis and meningitis.

Treatment of choice is endoscopic wide marsupialisation of the cyst and sometimes dacrocystorhinostomy tube placement to maintain patency.
